# Supplementary material for: Phototoxic Potential of Different DNA Intercalators for Skin Cancer Therapy: In Vitro Screening
Source: Int J Mol Sci. 2023 Mar 15;24(6):5602. doi: 10.3390/ijms24065602 (PMC10054552; doi:10.3390/ijms24065602)
Supplement: Supplementary file 1 [file ijms-24-05602-s001.zip › ijms-2204884-supplementary.pdf]

Supplementary materials

Phototoxic potential of different DNA-intercalators for skin cancer therapy: in vitro screening

Thais P. Pivetta [1,2], Tânia Vieira [3] Jorge C. Silva [3], Paulo A. Ribeiro [2], Maria Raposo [2,*]

1 CEFITEC, Department of Physics, NOVA School of Science and Technology, Universidade NOVA de Lisboa, 2829-516 Caparica, Portugal.
2 Laboratory of Instrumentation, Biomedical Engineering and Radiation Physics (LIBPhys-UNL), Department of Physics, NOVA School of Science and Technology, Universidade NOVA de Lisboa, 2829-516 Caparica, Portugal.
3 CENIMAT/I3N, Departamento de Física, Faculdade de Ciências e Tecnologia, Universidade Nova de Lisboa, 2829-516 Caparica, Portugal.
* Correspondence: mfr@fct.unl.pt (M.R.)

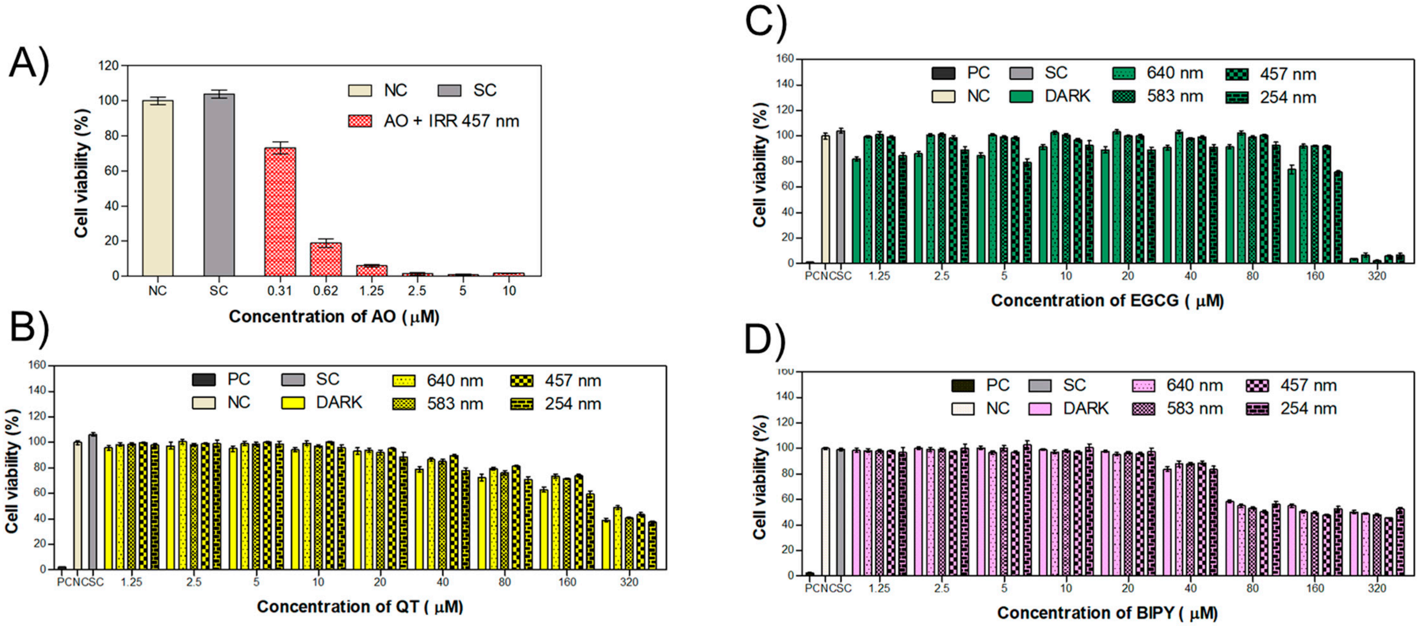

Supplementary Figure S1: (**A**) Cell viability of MET1 SCC cell line treated with AO and irradiated with blue light (457 nm). (**B**)–(**D**) cell viability of MET1 SCC cell line treated with QT, EGCG and BIPY, respectively. Samples kept in the dark were used as control compared to those submitted to different wavelengths irradiation. Values are mean ± combined stand-ard uncertainty (n=6). AO: acridine orange, QT: quercetin, EGCG: epigallocatechin gallate, BIPY: 2,2’-bipyridyl.

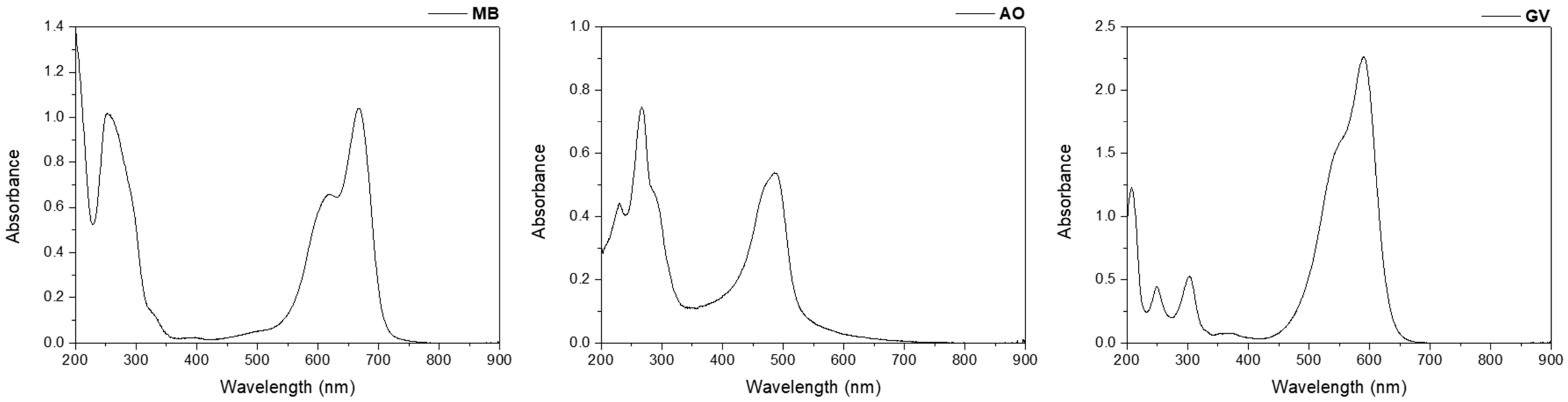

Supplementary Figure S2: Spectra of the dyes methylene blue (MB), acridine orange (AO) and gentian violet (GV).

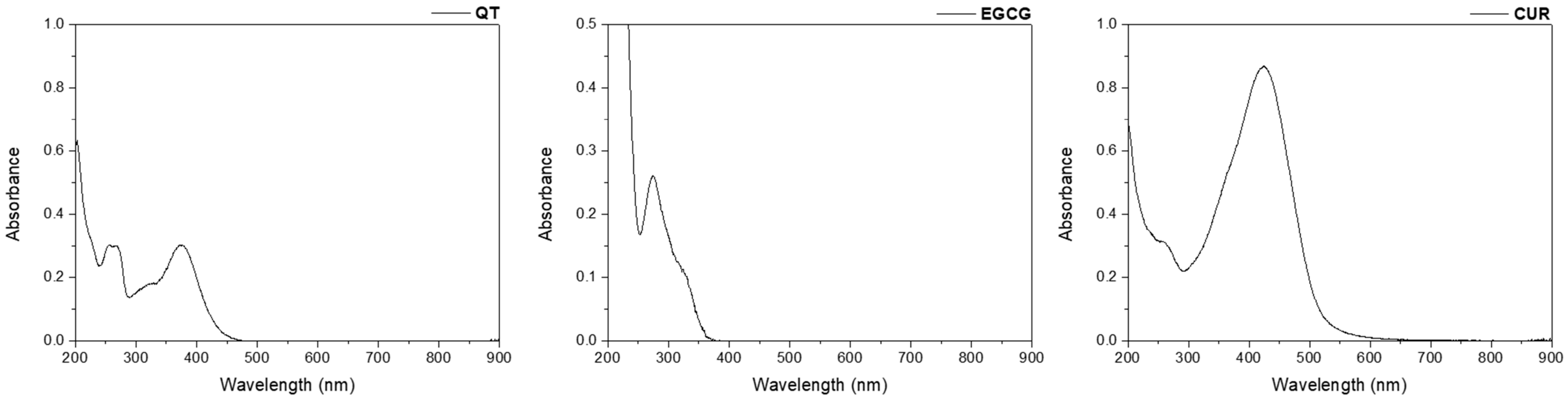

Supplementary Figure S3: Spectra of the natural products quercetin (QT), epigallocatechin-gallate (EGCG) and curcumin (CUR).

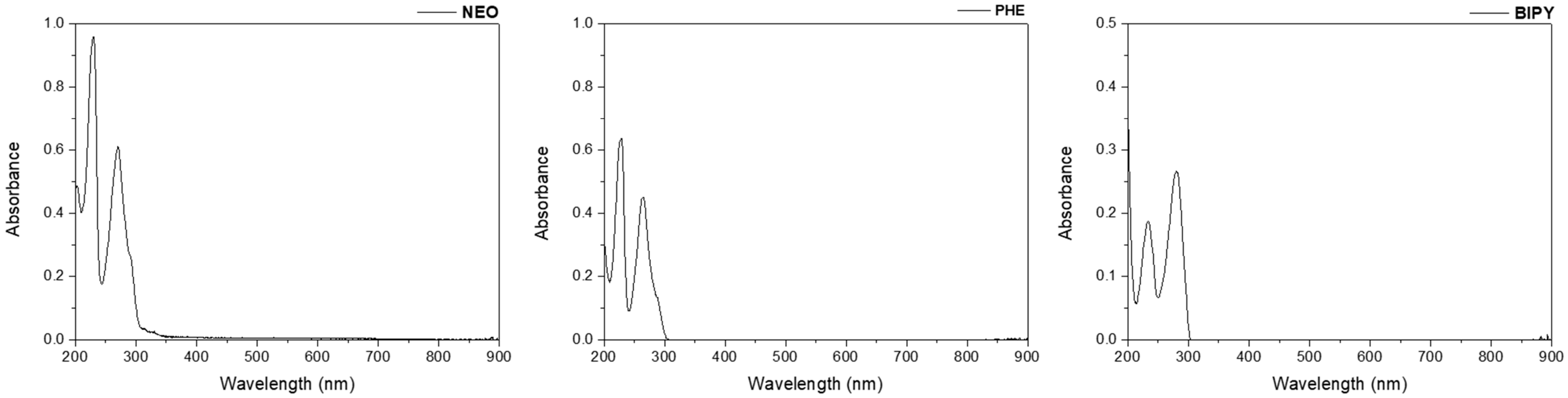

Supplementary Figure S4: Spectra of the chelating agents neocuproine (NEO), 1,10-phenanthroline (PHE) and 2,2′-bipyridyl (BIPY).
